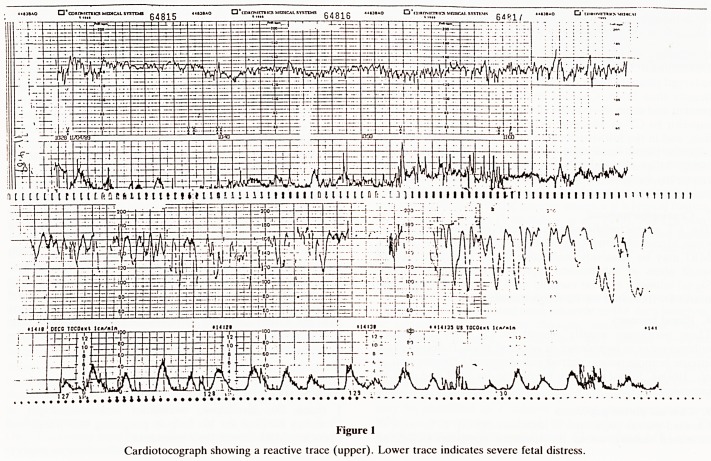# Plancental Abruption Following Vaginal Administration of Prostoglandin E2 for Induction of Labour

**Published:** 1990-12

**Authors:** R. Kulkarni, K. Hawkins, A. H. W. Boyle

**Affiliations:** North Devon District Hospital, Barnstaple, EX31 4JB; North Devon District Hospital, Barnstaple, EX31 4JB; North Devon District Hospital, Barnstaple, EX31 4JB


### Placental Abruption following Vaginal Administration of Prostaglandin E$_2$ For Induction of Labour

**R. Kulkarni**
**K. Hawkins**
**A. H. W. Boyle**
North Devon District Hospital, Barnstaple, EX31 4JB

The Prostaglandins PGE$_2$ and PGF$_2$ (Prostin, Upjohn) have now been available in clinical practice for over a decade. Prostaglandins are now the agents of choice for induction of labour if the cervix is unripe. Cervical ripening and the increase in compliance of the cervix induced by prostaglandin E$_2$ are believed to be a result of changes in the collagen and ground substance which are the two major components of the cervical stoma. However, the precise mode of action on the cervix is still unclear and this action is independent of their stimulant ction on uterine muscle. We have used vaginal pessaries (Prostin E$_2$) containing 2 mgs of Prostaglandin (prepared by our pharmacy) in patients for induction of labour, when the Bishop score was <7 (Bishop 1964) (1). Such usage of Prostin pessaries, under medical supervision, is generally unattended with any known major complications, either in the mother or the baby when given in this routine way.

We recently had to deal with an adverse incident in our hospital where administration of 4 milligrams of PGE$_2$ may have caused a significant placental abruption causing severe fetal distress in a previously uncompromised fetus.

### CASE REPORT

Mrs D.C., a 23 year old primigravida, was admitted for an elective induction of labour at the end of the 42nd week of pregnancy. She had an uneventful course in pregnancy until the 40th week when she had complained of bilateral ankle oedema but no other symptoms. She was briefly hospitalised for 24 hours for investigations and observation. Her blood pressure at this stage was 130/70 mg Hg. Her blood pressure at 16 weeks, at antenatal booking was 120/60 mm Hg. The following investigations were performed on her while she was in hospital:–24 hour urine, total protein 0.51 gram/litre and 0.8 gram/24 hours in a volume of 1640 ml. Haemoglobin 11.0 gram/dl, Platelet count $286 \times 10^9$/L, Serum electrolytes, serum urea and urate were within normal range. Cardiotographs showed normal reactive trace.

Her general condition and blood pressure remained stable and she returned for planned induction of labour.

On the day of induction of labour at 0600 hours, vaginal examination indicated an unfavourable cervix (Bishop score 3). PGE$_2$ $2 \times 2$ mg pessaries were inserted into the vaginal

**Figure 1**

Cardiotocograph showing a reactive trace (upper). Lower trace indicates severe fetal distress.

posterior fornix. A CTG for 20 minutes duration prior to the $PGE_2$ administration was normal and from then onwards fetal heart was monitored with intermittent auscultation.

About 5 hours following the insertion of the pessary, the patient started to complain of low abdominal discomfort and a CTG trace was applied, which was normal. Shortly afterwards the patient began to experience stronger contractions and also complained of vaginal loss of blood. She was transferred to the Labour Ward and a vaginal examination revealed that the cervix was 2 cms dilated and an artificial rupture of membranes produced hevily blood-stained liquor. The CTG recorded through the fetal scalp electrode showed an abnormal trace indicating severe fetal distress (figure 1) needing urgent delivery.

An emergency Caesarean Section was performed under general anaesthetic through a lower uterine segment incision. A live, severely asphyxiated female infant was delivered with an Apgar score of 1 at birth and 4 at 5 minutes. At delivery it was noted that the placenta, which was situated in the fundus, had undergone a major degree of separation involving two-thirds of its surface and the uterine cavity was full of blood clots (weighing approximately 700 gm). The remainder of the operation was completed in the usual way and the mother was transfused with 6 units of whole blood post operatively.

The child weighed 3900 gm at birth and had a cord haemoglobin of 10.0 gram/dl with raised fibrinogen degradation products indicating disseminated intravascular coagulation. Her condition was further complicated by renal failure. However, after two weeks her general condition started to improve with the improvement of renal function and she was discharged home. Subsequent follow up over 16 months has not indicated any residual complications in the mother or the child.

### DISCUSSION

This case illustrates the clear indication for continuous fetal monitoring after the administration of $PGE_2$ pessary in order to detect any serious complication such as placental abruption. We were interested to read in the medical literature a report showing a statistically significant association between placental abruption and vaginal administration of Prostaglandin $E_2$. Leund *et al.* (2) in their survey of 900 patients found placental abruption in 0.78% of patients who received 3 mg of $PGE_2$ vaginal pessary for induction compared to 0.06% in those who were not administered $PGE_2$. This difference was statistically significant. These authors also suggest that with their preliminary report, it would be worthwhile to conduct a large scale prospective study in establishing or disproving the statistically significant result that their retrospective study has shown. There have also been some reports of sudden fetal death associated with the use of Prostaglandin $E_2$ which could have been the result of premature placental separation (3).

Our case also emphasises the absolute need to administer the pessary at a time when an experienced obstetrician is available to deal with any untoward incident as happened in this particular case. It would seem politic not to administer $PGE_2$ overnight in a unit such as ours where a consultant has not got the assistance of a registrar for 8 months of the year and has to be on emergency call with only a junior trainee house officer (4).

Our case makes us also wonder whether there could be a small percentage of patients who may be hypersensitive to Prostaglandins and if so whether there are any useful clinical or laboratory tests in identifying this small but adversely reacting important group?

### REFERENCES

1. BISHOP, E. H. (1964) Pelvic Scoring for Elective Induction Obstet Gynaecol **24**, 266–268.
2. LEUNG, A., KWOK, P., and CHANG, A. (1987) Association between Prostaglandin $E_2$ and Placental ABruption. *Br. J. Obstet. Gynaecol.* **94**, 1001–1002.
3. STEWARD, P. and CALDER, A. A. (1981) Cervical Ripening (letter) *Br. J. Obstet. Gynaecol.* **88**, 1071–1072.
4. GREEN, J. M., COUPLAND, V. A., and KITZINGER, J. V. Observations on Obstetric Staffing: the Myth of the Three Tier Norm (1989) *Journal of Obstet. Gynaecol.* **9**, 289–292.

## Figures and Tables

**Figure 1 f1:**